# Diseases of the Skin: Their Description, Pathology, Diagnosis, and Treatment

**Published:** 1888-09

**Authors:** 


*Diseases of the Skin: their Description, Pathology, Diagnosis, and Treatment.* By H. RADCLIFFE CROCKER, M.D. (Lond.) With 76 Illustrations. London: H. K. LEWIS. 1888.

Another huge book on skin-diseases! In our last number we noticed a large volume on this subject, and now we are confronted with another of a similar kind containing over 700 pages. This time it is a reflex of the experience at University College Hospital. But as the practice there is much the same as at other hospitals, the existence of the book is not thereby justified. The fashion of producing books which are very much like those already existing is one that should be discouraged. The profession does not stand in need of treatises that are little else than expositions of routine practice, even although they bear the *imprimatur* of a department of a large London hospital. We view with dismay the threatening prospect of being invited to consider in a large monograph how, for example, they treat whitlow at St. Etheldreda's, and then, in a still larger volume, to find out that they do much the same thing at St. Silvester's.

It cannot be expected that we should read through such a volume as this before us. But a general look at it leads us to the belief that it is neither better nor worse than similar guides with which the profession is familiar. There was some hope that the modern tendency was towards simplicity in the classification of skin-diseases. We are disappointed at finding here a great inclination to minutely differentiate. Acne is resolved into twenty divisions. We have been teased over and over again with the unpractical divisions of eczema and psoriasis; but we did not expect to be plagued with twenty-three varieties of erythema, or fourteen of nævus. This may do for the unnecessary refinements of examination-purposes, but is a needless worry for the man in practice. The illustrations are of the usual character, and a useful list of formulæ

is given in an appendix. It is, however, distressing to find an author, who gives us etymological enlightenment about the derivation of words, writing about the "pubis."

The book is pleasant to read, because its literary style is so easy, and because there is much beauty about its mechanical finish.

